# Temporal and stimulus-specific transcriptional responses of maize to a biostimulant mixture of quillaja extract and fulvic acid

**DOI:** 10.1371/journal.pone.0360081

**Published:** 2026-10-08

**Authors:** Mohsen Hesami, Mohammad Rahbari, Ron A. Salzman

**Affiliations:** 1 Department of Plant Agriculture, University of Guelph, Guelph, ON, Canada; 2 BioLiNE^®^ Corporation, Alvinston, ON, Canada; 3 Department of Biochemistry and Biophysics, Texas A&M University, Texas, United States of America; Shiraz University, IRAN, ISLAMIC REPUBLIC OF

## Abstract

Biostimulant-induced responses in plants are dynamic, involving rapid signaling events followed by slower, downstream transcriptional and metabolic adjustments. To characterize the temporal molecular responses of maize to two biostimulant formulations, including fulvic acid (BLG) and mixture of fulvic acid and Quillaja extract (BLGQ), RNA sequencing was performed on leaf tissues collected at two time points representing the early (3 h) and later (27 h) transcriptional responses following biostimulant application. Differential gene expression, functional enrichment, and transcription factor analyses were subsequently conducted to compare the two treatments with untreated controls. Early responses revealed rapid activation of hormone signaling, ion transport, proteostasis, and transcription factor (TF) networks, with BLGQ inducing broader and stronger transcriptional changes compared to BLG. Notably, TF families such as *ERF*, *NAC*, *C2H2*, *WRKY*, and *MYB-related* were prominently upregulated, suggesting early transcriptional activation of genes associated withstress, growth, and developmental pathways. Later responses showed that BLG predominantly reinforced broad metabolic, redox, and transcriptional regulatory networks, whereas BLGQ emphasized signaling integration, vesicle trafficking, and secondary metabolism. Differential transcription factor modulation further highlighted stimulus- and time-specific strategies: BLG sustained upregulation of *MYB-related*, *NAC*, *GRAS*, and *ERF* TFs, while BLGQ selectively balanced upregulation and downregulation of *NF-YB*, *HSF*, and *bZIP* families to fine-tune signaling and specialized pathways. These transcriptomic results suggest that maize responses to biostimulants are highly dynamic and stimulus-specific, with BLG response associated with broader transcriptional changes in genes involved in general adaptive processes, whereas BLGQ response associated with more specific signaling and regulatory pathways. Our results provide transcriptomic insight into the temporal layers of biostimulant action and underline the importance of time-resolved analyses for understanding plant growth and stress adaptation.

## Introduction

Global agriculture faces increasing challenges associated with climate variability, declining soil quality, and the need to increase crop productivity while reducing environmental impacts [1]. These constraints have created a critical need to enhance crop productivity and sustainability using methods that are both efficient and environmentally responsible [2]. The use of plant biostimulants has emerged as a promising strategy for improving crop performance. Plant biostimulants enhance nutrient use efficiency, stimulate plant metabolic processes, and increase tolerance to abiotic stresses [3]. Among the diverse classes of biostimulants, fulvic acids have received considerable attention because of their ability to improve nutrient uptake, root development, antioxidant capacity, and stress adaptation [4,5]. More recently, plant-derived biostimulants containing saponins, particularly extracts from the *Quillaja saponaria* Molina tree, commonly referred to as Quillaja saponins, have attracted interest due to their bioactive properties and their potential to influence plant signaling, membrane function, and physiological responses [6].

Although numerous studies have demonstrated positive physiological effects of fulvic acids and other biostimulants, the molecular mechanisms underlying these responses remain only partially understood [7]. Recent advances in transcriptomic technologies, particularly RNA sequencing (RNA-seq), have enabled comprehensive characterization of plant responses at the genome-wide level by identifying differentially expressed genes, signaling pathways, metabolic networks, and transcription factors involved in biostimulant activity [8]. These approaches provide valuable opportunities to distinguish primary signaling events from downstream regulatory processes and to better understand how different biostimulant formulations influence plant growth and adaptation [9].

Despite increasing transcriptomic studies on plant biostimulants, several important knowledge gaps remain [8]. First, most investigations have focused on single biostimulant products, whereas little is known about the molecular responses induced by combinations of different biostimulant components [7], such as fulvic acids and *Quillaja* extracts. Second, transcriptomic responses are highly dynamic, yet relatively few studies have examined both early and later transcriptional responses following biostimulant application. Third, many transcriptomic investigations have been conducted under abiotic stress conditions, making it difficult to distinguish direct biostimulant-induced responses from transcriptional changes associated with stress alleviation. Consequently, the temporal molecular mechanisms underlying biostimulant activity under optimal growth conditions remain poorly understood, particularly in maize.

To address these knowledge gaps, we investigated the temporal transcriptomic responses of maize following foliar application of two biostimulant formulations: fulvic acid alone (BLG) and a combination of fulvic acid with *Quillaja saponaria* extract (BLGQ). Gene expression was analyzed at two time points representing early (3 h) and later (27 h) responses under optimal growth conditions using RNA sequencing. We hypothesized that both formulations would induce distinct temporal transcriptional programs associated with plant signaling, metabolism, growth, and stress-responsive pathways, and that the inclusion of *Quillaja* extract would produce broader and more specialized transcriptional responses than fulvic acid alone. The objective of this study was to characterize the dynamic molecular mechanisms underlying these responses and to provide new insights into the mode of action of these biostimulant formulations in maize.

## Materials and methods

### Plant growth conditions and experimental design

Maize (*Zea mays* L., cv. ‘Yellow Dent’) plants were grown individually in pots containing a natural sandy loam soil under greenhouse conditions at Texas A&M University. All pots received a uniform application of controlled-release fertilizer (Osmocote^®^ 15-9-12 NPK) at planting to ensure non-limiting nutrient availability, and plants were irrigated as needed to avoid moisture stress throughout the experiment. The experiment was conducted as a randomized complete block design (RCBD) with four independent experimental blocks (Fig 1). Each plot contained six plants per treatment, resulting in a total of 24 plants per treatment across the four blocks. Three treatments were evaluated: (i) untreated control (sprayed with deionized water containing 0.1% (v/v) Silwet surfactant without biostimulant), (ii) BioLiNE^^®^^ Gold fulvic acid (BLG) applied at an equivalent field rate of 1.0 L ha ^−1^, and (iii) a mixture of BioLiNE^^®^^ Gold fulvic acid and *Quillaja saponaria* extract (BLGQ; 1:1, w/w) applied at an equivalent field rate of 1.25 L ha ^−1^. Plants were harvested at two sampling times (3 h and 27 h after foliar application) to capture early and later transcriptional responses.

**Fig 1:**
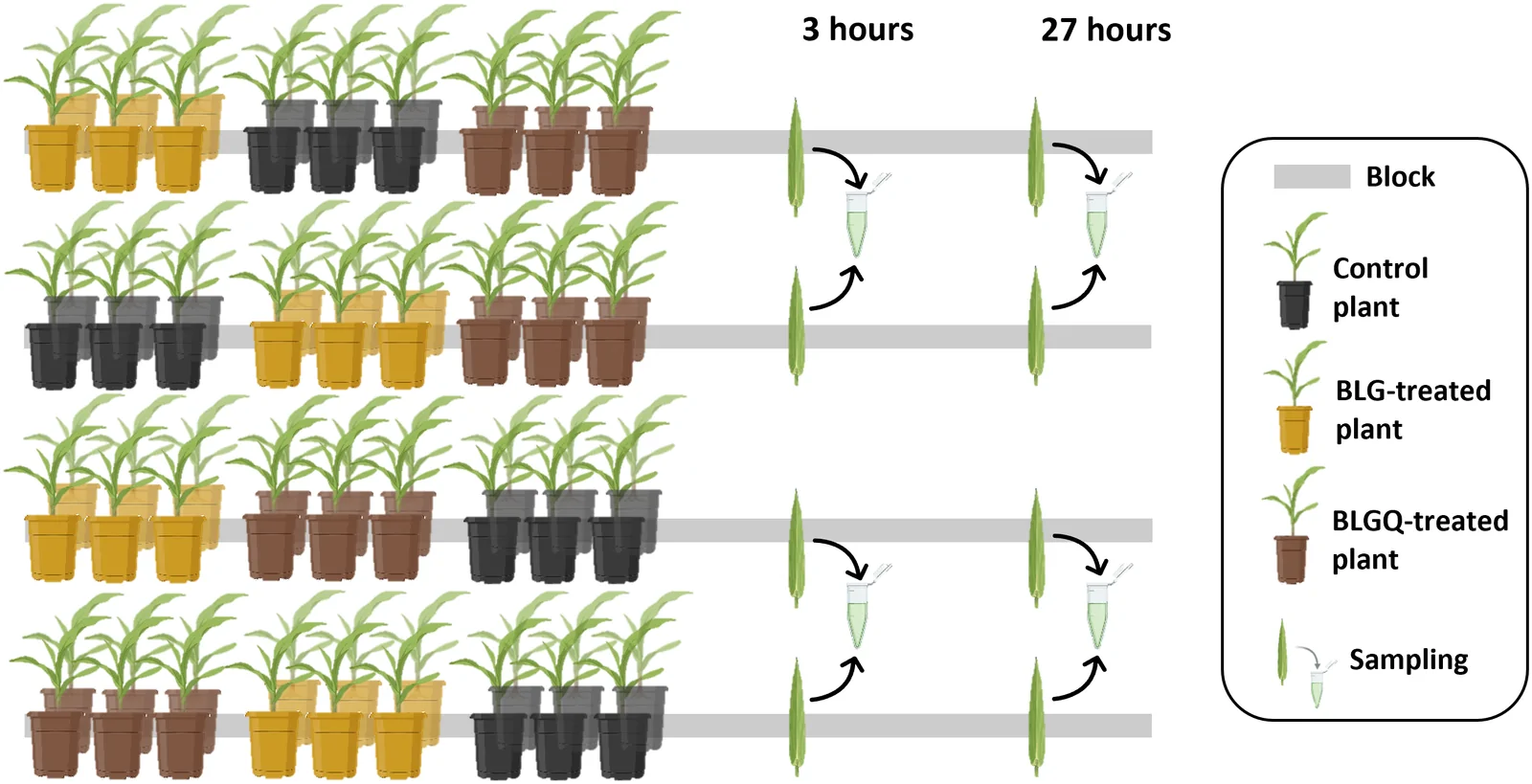
Schematic representation of the experimental design.

### Biostimulant preparation

The biostimulant samples were prepared by BioLiNE Corp. and shipped as blinded samples, to Texas A&M University for application in the trials. First samples consisted of BioLiNE^®^ Gold fulvic acid (FA). This product has 6% total dissolved solids (TDS) content, pH of ~2.2 and a hydrophobic fulvic acid (HFA) content of 2% as measured by the ISO 19822 method (Table 1). Foliar application of the BLG was made, at 3–4 leaf stage of the maize plants, at an effective rate of 1L/ha. The second sample consisted of a 1:1 (wt.:wt.) ratio of BioLiNE^®^ Gold fulvic acid and Plantae Labs (Renca, Chile) QL8 *Quillaja saponaria* extract. The QL8 contains >8% saponins, a TDS of 33%, and a pH of 4.1 (Table 1). Foliar application of BLGQ was made at 3–4 leaf stage of the maize plants, at an effective rate of 1.250 L/ha. To prepare the spray solutions, each of the two biostimulants were diluted in de-ionized water, assuming 46.8L/ha of water (5 USG/acre), thus, BLG was diluted to 1/46.8 ~ 2.13% and BLGQ to 1.25/46.8 ~ 2.67%. To improve wetting and ensure uniform coverage of the leaves, 0.1% v/v of Silwet surfactant was added to each spray solution. A hand-operated fine-droplet atomizer was used to spray 0.4 mL of spray solution per plant. For the control treatment, deionized water containing 0.1% (v/v) Silwet surfactant was applied using the same spray volume and application procedure as the biostimulant treatments, but without BLG or BLGQ.

**Table 1:** Physicochemical properties of BioLiNE^®^ Gold, *Quillaja saponaria* extract, and mixture of BioLiNE® Gold fulvic acid and *Quillaja saponaria* extract.

	QL8 *Quillaja saponaria* extract	BioLiNE^®^ Gold (BLG)	mixture of BLG and *Quillaja saponaria* extract (BLGQ)
pH	4.08	2.21	3.83
Electrical Conductivity (mS/cm)	13.93	48.00	27.13
Density	1.1395	1.0357	1.092
Total solids (TS, %)	36.17%	6.17%	19.89%
Ash (%)	5.23%	3.28%	4.15%
Organic Matter (OM, %)	30.94%	2.89%	15.74%
Total Organic Carbon (TOC, mg/L)	149910	6950	78000
Ratio of TOC: OM	0.49	0.24	0.5
Total Acidity (meq/g C)	3.85	17.14	5.28
Carboxyl Content (meq/g C)	1.65	14.9	3.19
Phenolic Content (meq/g C)	2.20	2.24	2.09
Metabolite Classes (% Contribution of each metabolite class) *			
Amino acids and derivatives	0.5	4.9	
Flavonoids	0.5	6.9	
Organic acids	4.8	46.4	
Nucleotides and derivatives	0.0	11.7	
Carbohydrates & derivatives	5.1	6.1	
Phenolic/polyphenolic compounds	87.0	24.0	
Other metabolites	2.1	0.0	

* Metabolite profiling was performed using a Thermo Scientific Ultimate 3000 UHPLC system coupled with a Q Exactive Orbitrap mass spectrometer. Samples were analyzed by reverse-phase and hydrophilic interaction liquid chromatography (RPC and HILIC) using heated electrospray ionization (HESI II) in both positive and negative ion modes with polarity switching.

### RNA extraction, library preparation, and sequencing

At the 3hr and 27hr timepoints (respectively) the shoot systems were cut off 4 cm above the soil surface. For each treatment and sampling time, shoot tissue from two plants within each of the four experimental blocks was collected, immediately wrapped in aluminum foil, frozen in liquid nitrogen, and stored at −80℃ until RNA extraction. Tissue from two plants in Blocks 1 and 2 was pooled to generate one composite biological replicate, while tissue from two plants in Blocks 3 and 4 was pooled to generate a second composite biological replicate. Thus, each treatment × sampling-time combination was represented by two pooled biological replicates, with each replicate consisting of tissue from four plants originating from two experimental blocks. Two technical replicates were generated for each biological replicate during the RNA-seq workflow. Tissues were kept frozen at -80C until processing for RNA extraction. Frozen tissue was ground to a fine powder, and total RNA was extracted using the RNeasy Plant Mini Kit (Qiagen) following the manufacturer’s protocol. RNA concentration and purity were assessed using a NanoDrop spectrophotometer (Thermo Fisher Scientific), and RNA integrity was verified with an Agilent 2100 Bioanalyzer. Only samples with RNA integrity number (RIN) ≥ 7.0 were used for library preparation. RNA-seq libraries were constructed using the Illumina TruSeq Stranded mRNA Library Prep Kit, following the manufacturer’s workflow for poly(A)-selected transcripts. Libraries were indexed, quantified with a Qubit fluorometer (Thermo Fisher Scientific), and pooled equimolarly. Sequencing was performed on an Illumina NovaSeq 6000 platform to generate 150-bp paired-end reads, targeting a minimum depth of 25 million reads per sample.

### RNA-Seq data processing and differential expression analysis

Raw sequencing reads were assessed for quality using FastQC and trimmed with Trimmomatic to remove low-quality bases and adapter sequences. Clean reads were aligned to the *Zea mays* B73 reference genome (AGPv5) using HISAT2 with default parameters. Alignment quality was evaluated with SAMtools, and gene-level read counts were generated using featureCounts (Subread package) based on the Ensembl maize gene annotation. Differential gene expression analysis was conducted using DESeq2 in R. Library size normalization was conducted using the median-of-ratios method implemented in DESeq2. Dispersion parameters were estimated using the DESeq2 generalized linear model framework. Statistical significance was determined using Wald tests, and P-values were adjusted for multiple testing using the Benjamini–Hochberg false discovery rate (FDR) correction. Genes with an adjusted p-value (FDR) < 0.05 and |log_2_ fold change| ≥ 1 were considered differentially expressed. Functional enrichment analyses, including Gene Ontology (GO) and Kyoto Encyclopedia of Genes and Genomes (KEGG) pathway enrichment, were performed using clusterProfiler. Transcription factor gene families were identified using PlantTFDB annotations. Venn diagrams, heatmaps, and volcano plots were generated in R using ggplot2 and VennDiagram packages. All scripts used in the analysis are available upon request.

## Results

### Global transcriptomic responses to biostimulant treatments

The application of BLG and BLGQ elicited clear temporal and treatment-specific changes in maize gene expression across the two sampling points. Comparative transcriptome profiling revealed that each biostimulant generated a distinct magnitude and pattern of transcriptional responses at 3 and 27 hours. Across all samples, gene expression profiles were broadly similar in magnitude, but each treatment induced a distinct subset of responsive genes. In the control condition, 17,776 genes were expressed (S1 Table). At 3 hours after BLG application, 17,360 genes (S1 Table) were expressed, of which 254 were differentially expressed (S2 Table). BLGQ treatment at the same time point resulted in expression of 16,846 genes (S3 Table), including 352 differentially expressed genes (S4 Table). At 27 hours, BLG-treated samples expressed 17,363 genes (S5 Table) with 228 showing differential expression (S6 Table), whereas BLGQ-treated samples expressed 18,675 genes (S7 Table) with 267 differentially expressed (S8 Table).

### Early transcriptional responses to biostimulant application

#### Differentially expressed genes following early biostimulant application.

At the 3-hour time point, BLG treatment resulted in 236 genes shared between BLG and control, along with 18 BLG-specific and 16 control-specific transcripts (Fig 2A, S2 Table). Under the same conditions, BLGQ triggered 332 shared differentially expressed genes, with an additional 20 genes unique to BLGQ and 22 unique to the control (Fig 2B, S4 Table). Gene expression profiling revealed that treatments with BioLiNE^®^ triggered distinct transcriptional responses (Fig 3A). Three hours after treatment, BLG induced 172 upregulated and 98 downregulated genes (Fig 3B), whereas BLGQ triggered 312 upregulated and 62 downregulated genes (Fig 3C).

**Fig 2:**
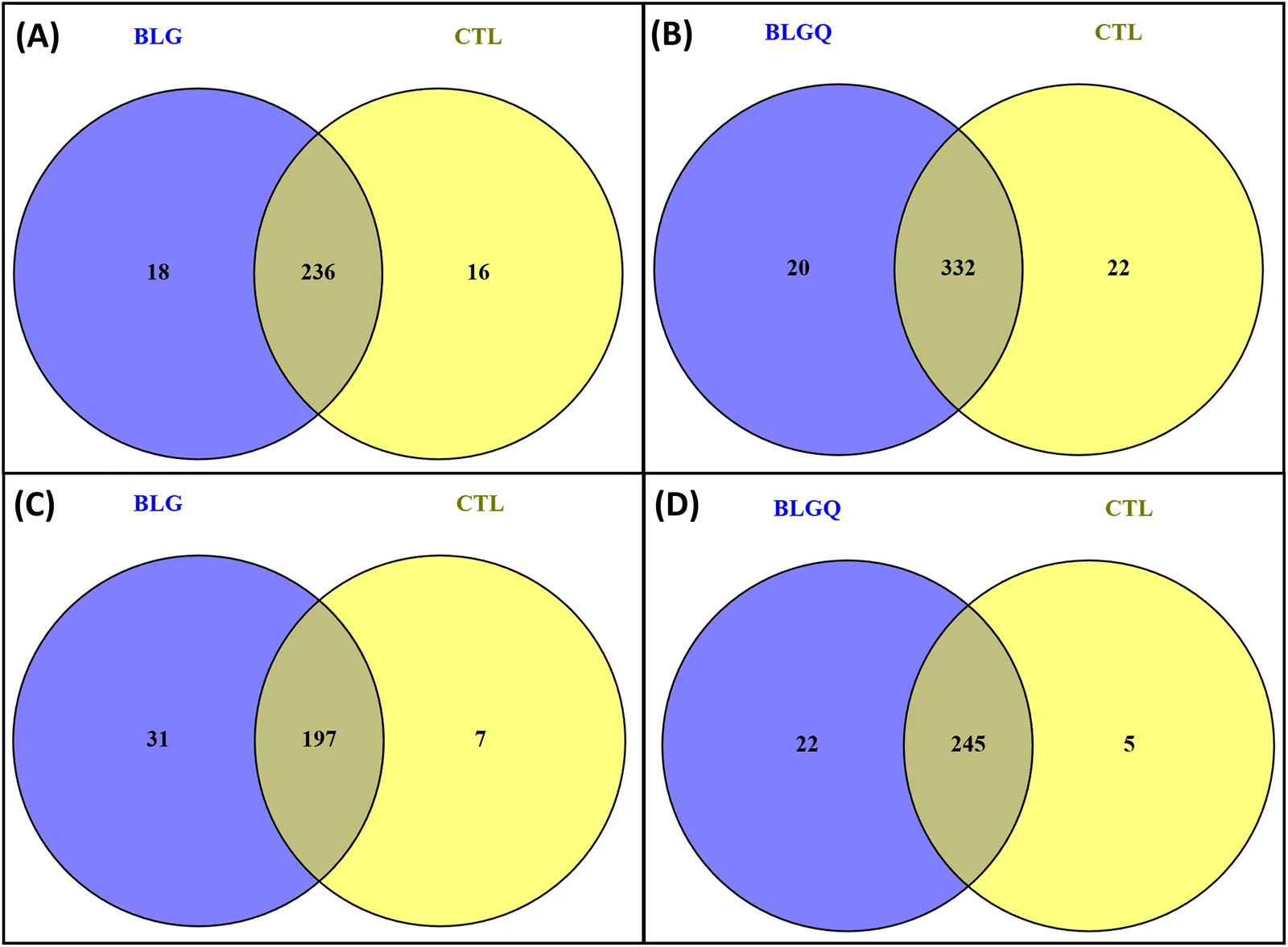
Venn diagram of differentially expressed genes of (A) BLG vs. CTL at 3 hours, (B) BLGQ vs. CTL at 3 hours, (C) BLG vs. CTL at 27 hours, (D) BLGQ vs. CTL at 27 hours.

**Fig 3:**
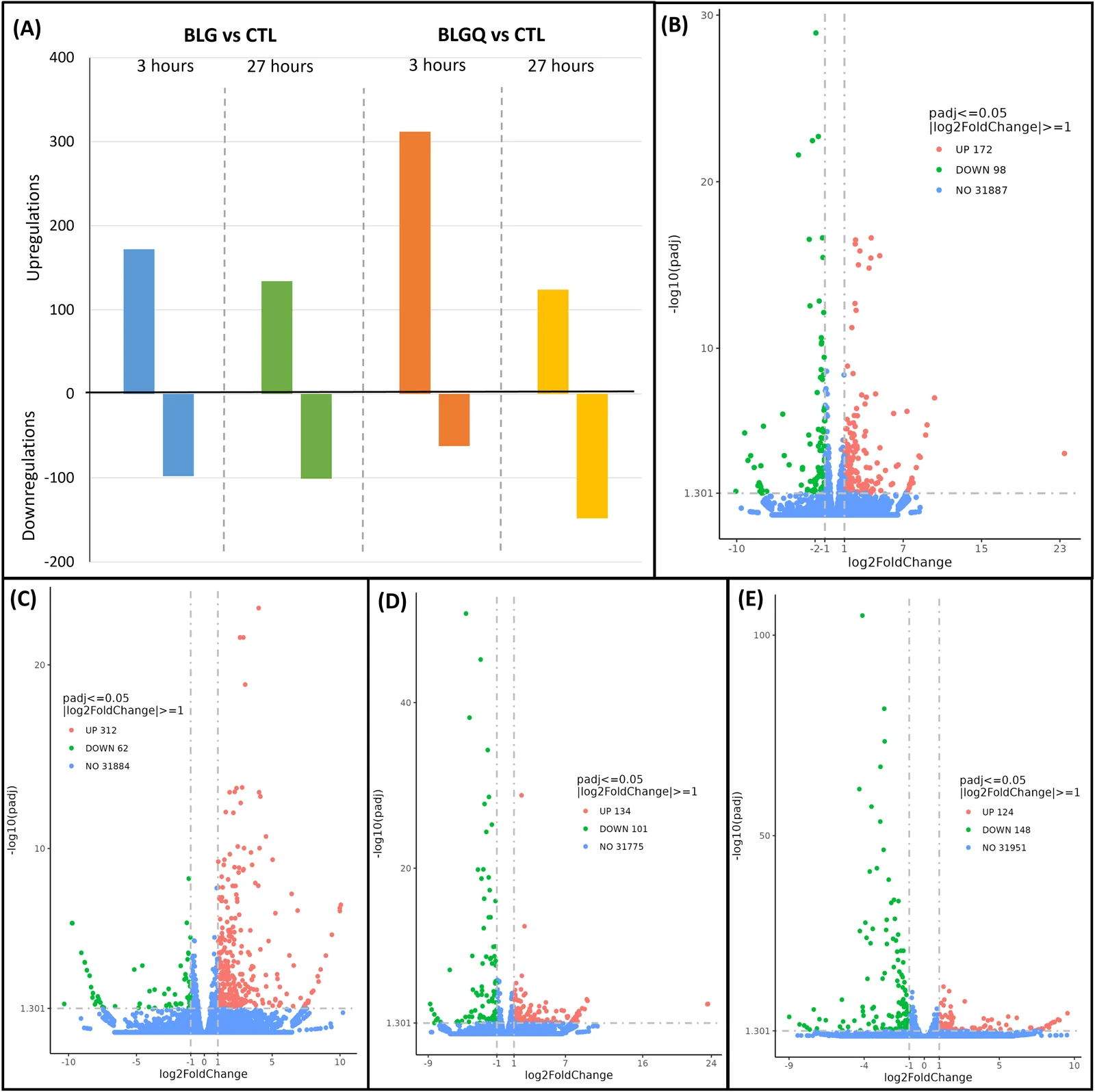
Differentially expressed genes statistics. (A) number of upregulated and downregulated genes, in the four comparisons (BLG vs. Control (CTL) at 3 hours and 27 hours; BLGQ vs. CTL at 3 hours and 27 hours), (B) Volcano plot of BLG vs. CTL at 3 hours, (C) Volcano plot of BLGQ vs. CTL at 3 hours, (D) Volcano plot of BLG vs. CTL at 27 hours, (E) Volcano plot of BLGQ vs. CTL at 27 hours.

#### Functional pathway enrichment following BLG treatment.

At the early response stage (3 hours post-BLG treatment), KEGG pathway analysis revealed enrichment in protein processing in the endoplasmic reticulum along with pathways related to amino acid biosynthesis, MAPK signaling pathway, endocytosis, spliceosome function, pantothenate and CoA biosynthesis, arginine and proline metabolism, plant–pathogen interactions, 2-oxocarboxylic acid metabolism, and plant hormone signal transduction (Fig 4A).

**Fig 4:**
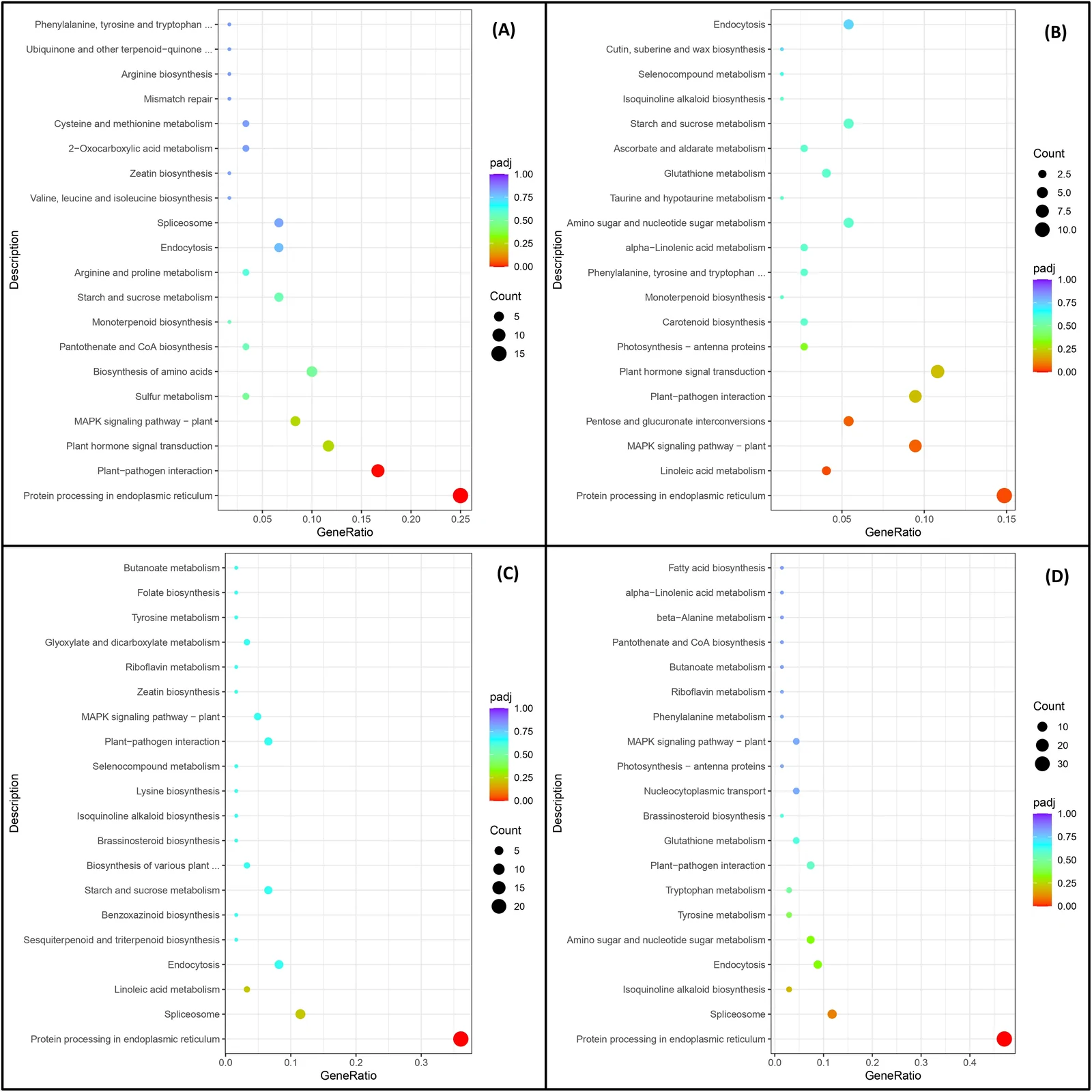
KEGG enrichment of differentially expressed genes in (A) BLG vs. CTL at 3 hours, (B) BLGQ vs. CTL at 3 hours, (C) BLG vs. CTL at 27 hours, (D) BLGQ vs. CTL at 27 hours.

#### Transcription factor responses following BLG treatment.

In addition, 36 transcription factor classes exhibited differential expression at 3 hours following BLG treatment (Fig 5A). Among up-regulated transcription factors, ERF was the most prominent class with 19 genes, followed by C2H2, NAC, and WRKY, each with 11 genes. In contrast, MYB-related genes were the most abundant among down-regulated transcription factors (8 genes), followed by bZIP (6 genes) (Fig 5A).

**Fig 5:**
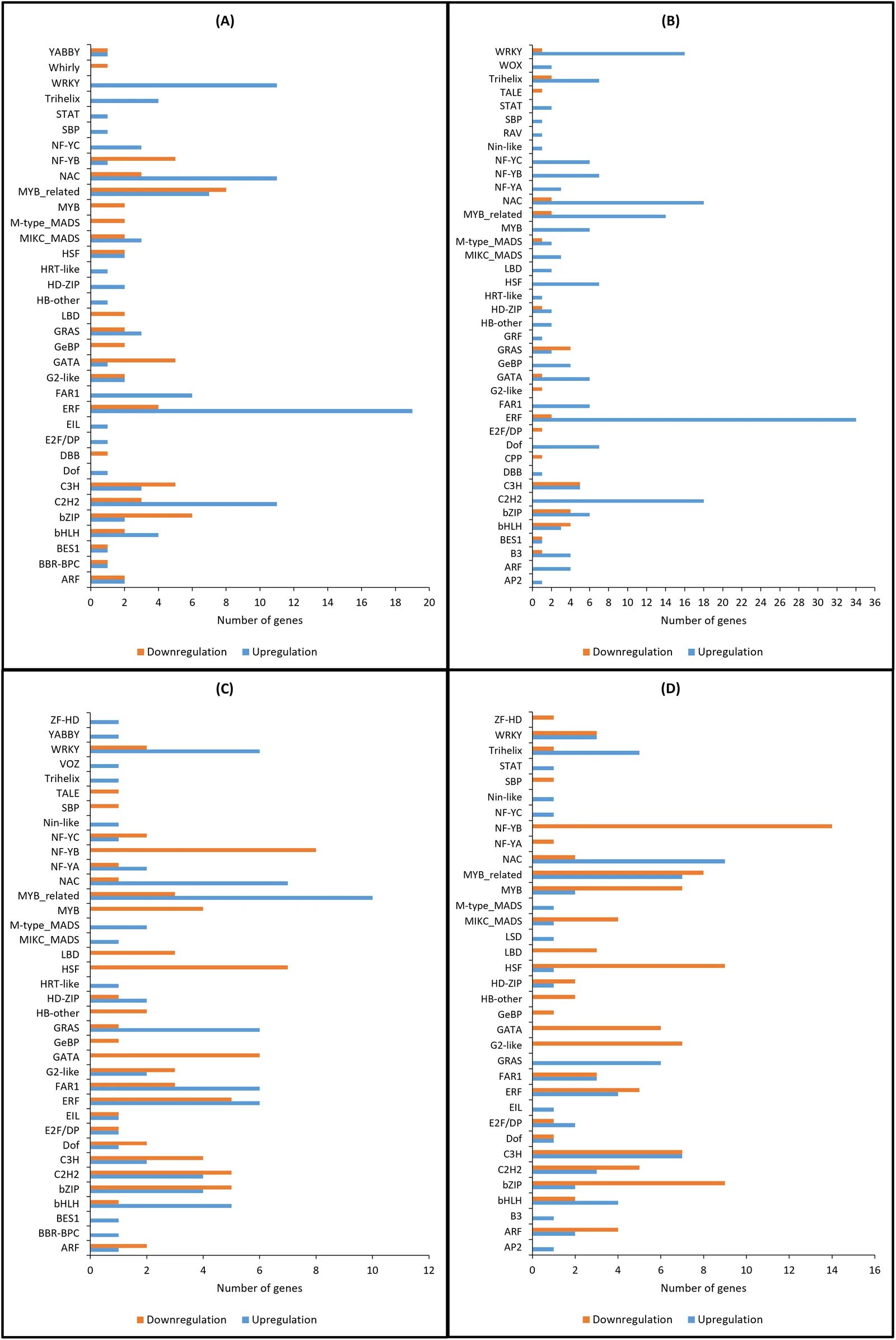
Number of transcription factor classes of differentially expressed genes in (A) BLG vs. CTL at 3 hours, (B) BLGQ vs. CTL at 3 hours, (C) BLG vs. CTL at 27 hours, (D) BLGQ vs. CTL at 27 hours.

#### Functional pathway enrichment following BLGQ treatment.

At the early response stage (3 hours post-BLGQ treatment), KEGG pathway analysis revealed enrichment in protein processing in the endoplasmic reticulum along with pathways related to MAPK signaling pathway, plant–pathogen interactions, linoleic acid metabolism, and plant hormone signal transduction (Fig 4B).

#### Transcription factor responses following BLGQ treatment.

Forty transcription factor classes exhibited differential expression at 3 hours following BLGQ treatment (Fig 5B). Among up-regulated transcription factors, ERF was the most prominent class with 34 genes, followed by C2H2 and NAC each with 18 genes. In contrast, C3H were the most abundant among down-regulated transcription factors (5 genes), followed by bHLH, bZIP, and GRAS each with 4 genes (Fig 5B).

### Later transcriptional responses to biostimulant application

#### Differential gene expression following late biostimulant application.

At the 27-hour time point, BLG treatment resulted in 197 genes shared between BLG and control, along with 31 BLG-specific and 7 control-specific transcripts (Fig 2C, S6 Table). Under the same conditions, BLGQ triggered 245 shared differentially expressed genes, with an additional 22 genes unique to BLGQ and 5 unique to the control (Fig 2D, S8 Table). At 27 hours post-treatment, BLG led to 134 upregulated and 101 downregulated genes (Fig 3D), whereas BLGQ caused 124 upregulated and 148 downregulated genes (Fig 3E). These results indicate a temporal shift in gene expression regulation. At 3 hours post-treatment, the proportion of upregulated to downregulated genes was greater for both BLG and BLGQ than at 27 hours, indicating that downregulation becomes more prominent as a late response to the application of biostimulants (Fig 3A).

#### Functional responses to BLG at 27 h.

At the later response stage (27 hours post-BLG treatment), KEGG pathway analysis indicated enrichment in protein processing in the endoplasmic reticulum as well as pathways related to spliceosome, endocytosis, biosynthesis of secondary metabolites, and starch and sucrose metabolism (Fig 4C). At 27 hours post-BLG treatment, 37 transcription factor classes exhibited differential expression (Fig 5C). Among up-regulated transcription factors, MYB-related was the most prominent class with 10 genes, followed by NAC (7 genes), and GRAS, ERF, and FAR1, each with 6 genes. In contrast, NF-YB was the most abundant among down-regulated transcription factors (8 genes), followed by HSF (6 genes) (Fig 5C).

#### Functional responses to BLGQ at 27 h.

For BLGQ, the late response (27 hours post-treatment) also showed enrichment in protein processing in the endoplasmic reticulum, but additional pathways were observed, including the spliceosome, isoquinoline alkaloid biosynthesis, endocytosis, amino sugar and nucleotide sugar metabolism, plant–pathogen interactions, tyrosine metabolism, and indole alkaloid biosynthesis (Fig 4D). At 27 hours post-BLGQ treatment, 35 transcription factor classes were differentially expressed (Fig 5D). NAC was the most prominent among up-regulated transcription factors (9 genes), followed by MYB-related and C3H, each with 7 genes. Among down-regulated transcription factors, NF-YB was the most abundant (14 genes), followed by bZIP and HSF, each with 9 genes (Fig 5D).

## Discussion

Biostimulant-induced responses are inherently dynamic, involving rapid signaling events followed by slower, downstream adjustments, and failing to distinguish between these phases has been a major weakness in many previous studies [10]. Early transcriptional changes typically reflect the plant’s immediate perception of the biostimulant, including activation of receptor-mediated pathways, ion fluxes, kinase signaling, and transcription factors that set the trajectory for later physiological outcomes [11]. In contrast, later responses integrate the cumulative effects of these initial signals and manifest as altered metabolic activity, resource allocation, or cellular remodeling [11]. Without resolving these temporal layers, it is impossible to determine whether a biostimulant acts primarily by triggering early signaling pathways, by modulating downstream metabolism, or by inducing prolonged regulatory shifts [12]. Time-resolved analyses therefore provide a more mechanistic and biologically coherent understanding of biostimulant function and are essential for interpreting how complex formulations influence plant physiology [13].

The transcriptomic profiling of maize tissue 3 hours after BLG (fulvic acid-containing biostimulant) application revealed a distinct set of genes uniquely induced by the treatment, indicating an early and specific molecular response even under optimal growth conditions.

Among the 18 BLG-exclusive genes, several functional categories stand out. Hormone-related genes, such as the auxin-responsive IAA20 and the rapid alkalinization factor RALF, suggest that BLG was associated with rapid transcriptional changes in genes involved in growth-related signaling pathways [14]. IAA20, an AUX/IAA family repressor, implies fine-tuning of auxin-mediated transcriptional programs [15], while RALF peptides are known to influence cell expansion and early stress or signaling responses [16]. Membrane transport and water homeostasis are also targeted, as evidenced by the induction of aquaporin TIP3−1 and sodium/hydrogen exchanger NHX2 [8,17], indicating that BLG induced transcriptional changes in genes associated with nutrient and ion transport even in the absence of environmental stress. Additionally, genes related to energy metabolism and redox regulation (e.g., mitochondrial alternative oxidase AOX1B and NUDIX hydrolase 14) suggest that BLG induces transcriptional changes in genes associated with metabolic regulation and redox homeostasis, potentially preparing the plant for accelerated growth [12,18]. Developmental regulators MAD2 and PDF1 further imply that BLG altered the expression of genes previously associated with cell division and tissue differentiation [19]. In addition, 6 genes with uncharacterized functions (e.g., Zm00001eb173110, Zm00001eb121720, Zm00001eb157670, Zm00001eb057930, Zm00001eb077680, and Zm00001eb240710) are also induced, suggesting the presence of novel regulatory pathways triggered by BLG that warrant further investigation [9].

In contrast, the 16 genes uniquely expressed in control plants represent baseline maintenance and housekeeping functions under optimal conditions. Receptor-like kinases (PERK1, WNK2), cell wall modulators (Expansin-B5, FH11), and cytoskeletal/structural proteins (MOR1, NUP43) appear to sustain constitutive growth, cellular organization, and signal perception [11]. Their absence in BLG-treated plants indicates that early transcriptional resources are temporarily redirected away from baseline maintenance toward more proactive pathways promoting growth, nutrient transport, and developmental priming [9].

Transcriptomic profiling 3 hours after application of BLGQ (fulvic acid combined with Quillaja saponins) revealed a total of 20 genes that were exclusively detected in BLGQ-treated plants, highlighting multiple functional themes. Proteostasis and signaling regulation emerge as key responses. The presence of BPA1, a proteasome-associated protein, and two U-box E3 ubiquitin ligases (PUB21, PUB23) indicates early engagement of protein turnover and post-translational regulation, likely enabling selective degradation or modification of regulatory proteins to fine-tune signaling [20]. The detection of protein phosphatase 2C (PP2C37) further supports the idea that BLGQ modulates reversible phosphorylation events, potentially adjusting kinase-mediated signaling cascades in response to the biostimulant [21].

Ion transport and nutrient homeostasis appear prominently [22]. Plasma membrane calcium ATPase ACA2, the S-type anion channel SLAH2, and copper transporter COPT1 suggest that BLGQ rapidly affects cation and anion fluxes, as well as micronutrient transport [22]. These transcriptional changes may reflect early molecular responses associated with nutrient transport and ion homeostasis, even without external stress, which could contribute to enhanced metabolic efficiency [23]. Transcriptional and metabolic regulation is also evident. Induction of the transcription factor bZIP19, long-chain base biosynthesis protein LCB1B, and enzymes such as aldose reductase and GDSL esterase/lipase LTL1 points to early modulation of hormone, lipid, and carbohydrate metabolism [24]. These changes may be involved in transcriptional regulation of cellular metabolism [12,13]. Several genes with less-characterized functions may represent novel regulatory pathways activated by BLGQ. Their presence underscores the complexity and specificity of the early transcriptional response. These results suggest that BLGQ triggers a distinct early transcriptomic signature in maize that diverges from both untreated controls and BLG-alone treatments, reflecting the additive or synergistic effects of fulvic acid and Quillaja saponins.

BioLiNE^®^ treatments (i.e., BLG and BLGQ) are associated with transcriptional changes involving signaling, transport, and metabolic processes, potentially explaining reported improvements in growth and resource efficiency [3,12,25]. The concurrent activation of proteostasis machinery, signaling regulators, and metabolic enzymes suggets that biostimulant treatments prime maize cells, creating a molecular environment favoring early growth and development [10].

Transcriptomic analysis 3 hours post-application reveals that both BLG and BLGQ induce rapid and extensive changes in transcription factor (TF) expression, but the magnitude and breadth of responses differ markedly, suggesting distinct early regulatory strategies. Both treatments prominently upregulate TF families involved in signaling integration, stress anticipation, and hormonal crosstalk. ERF, C2H2, NAC, WRKY, and MYB-related TFs are consistently activated, highlighting early engagement of modules typically associated with reactive oxygen species signaling, cell wall integrity sensing, and hormone-mediated transcriptional control [9,26]. In BLG, ERF and NAC each show 19 and 11 upregulated genes, whereas in BLGQ these numbers increase substantially to 34 and 18, respectively, suggesting that the addition of Quillaja saponins amplifies early signaling and is associated with a broader transcriptional response. Similarly, C2H2 zinc-finger TFs increase from 11 upregulated genes in BLG to 18 in BLGQ, reinforcing the idea that BLGQ exerts a broader regulatory influence.

BLGQ induces stronger upregulation in hormone-responsive and growth-associated TF families, including ARF, B3, bZIP, GATA, Dof, and NF-Y subunits. While BLG triggers modest shifts (e.g., ARF: 2 up / 2 down; bZIP: 2 up / 6 down), BLGQ drives more pronounced activation (ARF: 4 up; bZIP: 6 up / 4 down), suggesting enhanced early modulation of auxin, ABA, and other hormone pathways. Notably, developmental regulators such as MIKC_MADS, M-type MADS, GRF, and WOX show modest but consistent activation under BLGQ, whereas BLG elicits only minimal changes. This pattern implies that BLGQ may initiate transcriptional programs linked not only to stress signaling but also to early growth and differentiation processes, potentially providing a dual biostimulatory effect [13].

Downregulation across several TF families (e.g., bHLH, bZIP, C3H, GATA, NF-YB, GRAS) is observed in both treatments, although BLG shows relatively more repression in housekeeping-related TFs (e.g., bZIP: 6 down; NF-YB: 5 down). BLGQ exhibits fewer downregulated genes in the same categories, suggesting that the addition of Quillaja may mitigate the transient suppression of baseline homeostatic TFs, allowing a more balanced early regulatory state while still promoting signaling and metabolic priming.

Taken together, these patterns indicate that BLG triggers an early “alert” state in maize, selectively activating signaling and hormone-related TFs while transiently downregulating housekeeping regulators [27]. The addition of Quillaja (BLGQ) amplifies this early response, broadening the spectrum of TFs engaged and promoting a more balanced activation of genes associated with growth- and stress-related processes. This dual pattern may underlie the observed physiological benefits of BLGQ, representing a combination of rapid signaling priming and transcriptional regulation of developmental pathways rather than a simple stress-response effect [13].

The Venn diagram analysis of maize responses at 27 hours post-treatment with BLG and BLGQ revealed both overlapping and distinct transcriptional signatures, reflecting partially convergent but stimulus-specific regulatory pathways [12]. The 31 genes uniquely regulated by BLG include those involved in amino acid metabolism (ALD1), energy metabolism (L-lactate dehydrogenase), calcium signaling (CML19, CBL7), redox regulation (GRXS1), ethylene signaling (ERF109), receptor-like kinases (PERK1, LRR-RLK), chromatin remodeling (SWI3B), and carbohydrate metabolism (GOLS1). These late transcriptional changes suggest that BLG maintains engagement of core metabolic and regulatory networks, sustaining stress adaptation, growth modulation, and transcriptional reprogramming [7,28]. The presence of calcium-binding and receptor-like kinase genes indicates prolonged activation of calcium-mediated signaling and extracellular perception, often linked to hormonal crosstalk and late stress responses [29–31].

The 22 genes uniquely modulated by BLGQ include AP2-like ethylene-responsive transcription factor CRL5, cytochrome P450s, secretory carrier proteins (SCAMP4), phosphoinositide kinases (PIPK1), glutaredoxins (GRXS1), and dioxygenases (DAO). This profile indicates that BLGQ primarily influences late-stage signaling and specialized metabolic pathways. The induction of phosphoinositide kinase and vesicle-trafficking genes suggests a focus on membrane-mediated signaling and intracellular transport, supporting late-phase stress adaptation and secondary metabolite regulation [32,33].

Some genes, such as GRXS1 and CYP72A15, are regulated by both BLG and BLGQ, highlighting conserved late-response modules in redox balance and secondary metabolism [34]. However, BLG predominantly reinforces broad metabolic and transcriptional regulatory programs, whereas BLGQ emphasizes signaling, vesicle trafficking, and specialized metabolic responses. This divergence suggests that, at 27 hours, BLG was associated with differential expression of genes involved in metabolic and regulatory pathways, while BLGQ fine-tunes signaling networks and metabolic specialization.

Analysis of TF expression further supports these patterns. BLG induces broad transcriptional reprogramming, with pronounced upregulation of MYB_related (10 genes), GRAS (6), NAC (7), ERF (6), FAR1 (6), and WRKY (6) genes, which are central to stress signaling, hormone responsiveness, and developmental regulation [12]. Mixed regulation in families such as bZIP, C2H2, and G2-like suggests fine-tuning of specific TF members for precise downstream control [9]. Downregulation is most evident in GATA (6), bZIP (5), and HSF (7), indicating selective suppression of certain growth- or stress-related programs [13].

BLGQ, in contrast, shows stronger repression in TF families such as NF-YB (14 downregulated), bZIP (9), and HSF (9), while still upregulating key regulators including NAC (9), MYB_related (7), GRAS (6), Trihelix (5), and AP2 (1). This pattern suggests an emphasis on late-stage signaling, transcriptional integration, and specialized regulatory pathways, rather than broad metabolic control [27]. Conserved TF families (NAC, GRAS, ERF, MYB_related) are modulated by both treatments, but the magnitude and direction of regulation differ, with BLG favoring sustained activation of metabolic and stress-related modules, and BLGQ selectively balancing activation and repression to orchestrate signaling, vesicle trafficking, and secondary metabolism.

At 27 hours, maize exhibits stimulus-specific transcriptional programming in response to BLG and BLGQ. BLG maintains broad metabolic, stress-responsive, and transcriptional networks, whereas BLGQ selectively modulates TFs and other genes to fine-tune signaling, developmental, and secondary metabolite pathways. Together, these responses underscore the complementary and nuanced roles of BLG and BLGQ in orchestrating late adaptive responses in maize [10].

Comparing early and late transcriptional responses highlights the dynamic progression of maize adaptation to biostimulant treatments [11]. Early responses at 3 hours are dominated by rapid signaling events, including activation of hormone-responsive genes, ion transporters, and transcription factors, which collectively prime the plant for subsequent physiological adjustments [11]. BLG primarily triggers early growth- and stress-related TFs, setting an early transcriptional response, whereas BLGQ amplifies and broadens this early response, engaging additional regulatory pathways, proteostasis mechanisms, and nutrient transport processes. This indicates that the presence of Quillaja saponins in BLGQ strengthens and balances early signaling, promoting transcriptional responses associated with stress-related and developmental pathways [13].

Later responses at 27 hours reflect the integration of these initial signals into more specialized and sustained transcriptional programs. BLG reinforces broad metabolic, stress, and transcriptional networks, maintaining energy, amino acid, and redox homeostasis, while BLGQ shifts emphasis toward signaling integration, vesicle trafficking, and secondary metabolism. Differences in transcription factor regulation mirror this pattern: BLG sustains upregulation of MYB-related, NAC, GRAS, and ERF families to support generalized adaptive pathways, whereas BLGQ selectively balances activation and repression of NF-YB, HSF, bZIP, and other TFs to fine-tune signaling and developmental programs. Together, these temporal and treatment-specific differences underscore that maize responses evolve from early perception and priming toward stimulus-specific late adaptation [10], with BLG driving general preparedness and BLGQ coordinating targeted, specialized pathways.

Our transcriptomic analysis reveals that BLG and BLGQ treatments induce distinct but overlapping responses in maize, highlighting both early (3 h) and late (27 h) phases of gene regulation. Across both treatments, key functional categories (i.e., photosynthesis, transcriptional regulation, hormone signaling, stress response, and secondary metabolism) were prominently affected, suggesting a coordinated adaptation to these stimuli [12].

By 3 h, BLG treatment triggered rapid modulation of transcription factors (e.g., MYB, NAC, WRKY families) and signaling components, including kinases and calcium-related proteins. This early activation likely reflects an early transcriptional activation of regulatory networks necessary for downstream metabolic and stress responses [9]. In parallel, genes involved in photosynthesis, such as chloroplastic pentatricopeptide repeat-containing proteins and phosphoenolpyruvate translocators were upregulated, possibly indicating a transient stimulation of energy metabolism to support defense and growth adaptation [26]. Notably, BLGQ elicited a slightly different early signature, with selective upregulation of flowering-related and cell-wall biosynthesis genes, suggesting that BLGQ may preferentially activate developmental reprogramming alongside stress signaling [6,13].

By 27 h, BLG treatment resulted in widespread transcriptional changes affecting secondary metabolism, redox homeostasis, and hormone pathways, including ethylene, ABA, and auxin signaling. Upregulation of genes involved in cytochrome P450 activity and phenylpropanoid biosynthesis suggests transcriptional enrichment of genes involved in detoxification-related pathways and secondary metabolite production, likely as a protective mechanism [35,36]. BLGQ, in contrast, maintained strong expression of transcriptional regulators, including MYB- and GRAS-domain proteins, and genes controlling growth pattern and cell determination, implying sustained developmental modulation [12]. Additionally, stress-related proteins, including glutathione transferases and thioredoxins, remained upregulated under BLGQ, highlighting a prolonged defense response relative to BLG [13].

While both BLG and BLGQ activated common stress and metabolic pathways, the magnitude and timing differed. BLG provoked a rapid but transient metabolic and transcriptional surge that shifted towards secondary metabolism and defense at later time points [4]. BLGQ induced more persistent changes in transcription factors, cell-wall, and developmental genes, suggesting that it integrates stress signaling with growth regulation more strongly than BLG. The differential modulation of hormone signaling components (e.g., auxin-related SAUR and GH3 genes) further supports the notion that BLGQ may contribute to transcriptional regulation of growth-related pathways under stress [13], whereas BLG primarily triggers immediate defense responses.

The early upregulation of photosynthetic and energy-related genes in both treatments may support energy metabolism. and reducing power to support transcriptional reprogramming and metabolite synthesis [37]. The sustained activation of transcription factors and developmental regulators under BLGQ may contribute to transcriptional regulation of growth- and defense-related pathways, optimizing survival while maintaining developmental progression. The observed induction of cytochrome P450s, glutathione transferases, and redox enzymes highlights a core oxidative stress mitigation mechanism common to both treatments, essential for preserving cellular integrity under stress [38,39]. Together, these findings demonstrate that BLG and BLGQ elicit both shared and unique transcriptional programs in maize (Fig 6). BLG predominantly activates rapid stress-responsive and metabolic pathways, whereas BLGQ coordinates stress responses with developmental and growth-regulatory programs.

**Fig 6:**
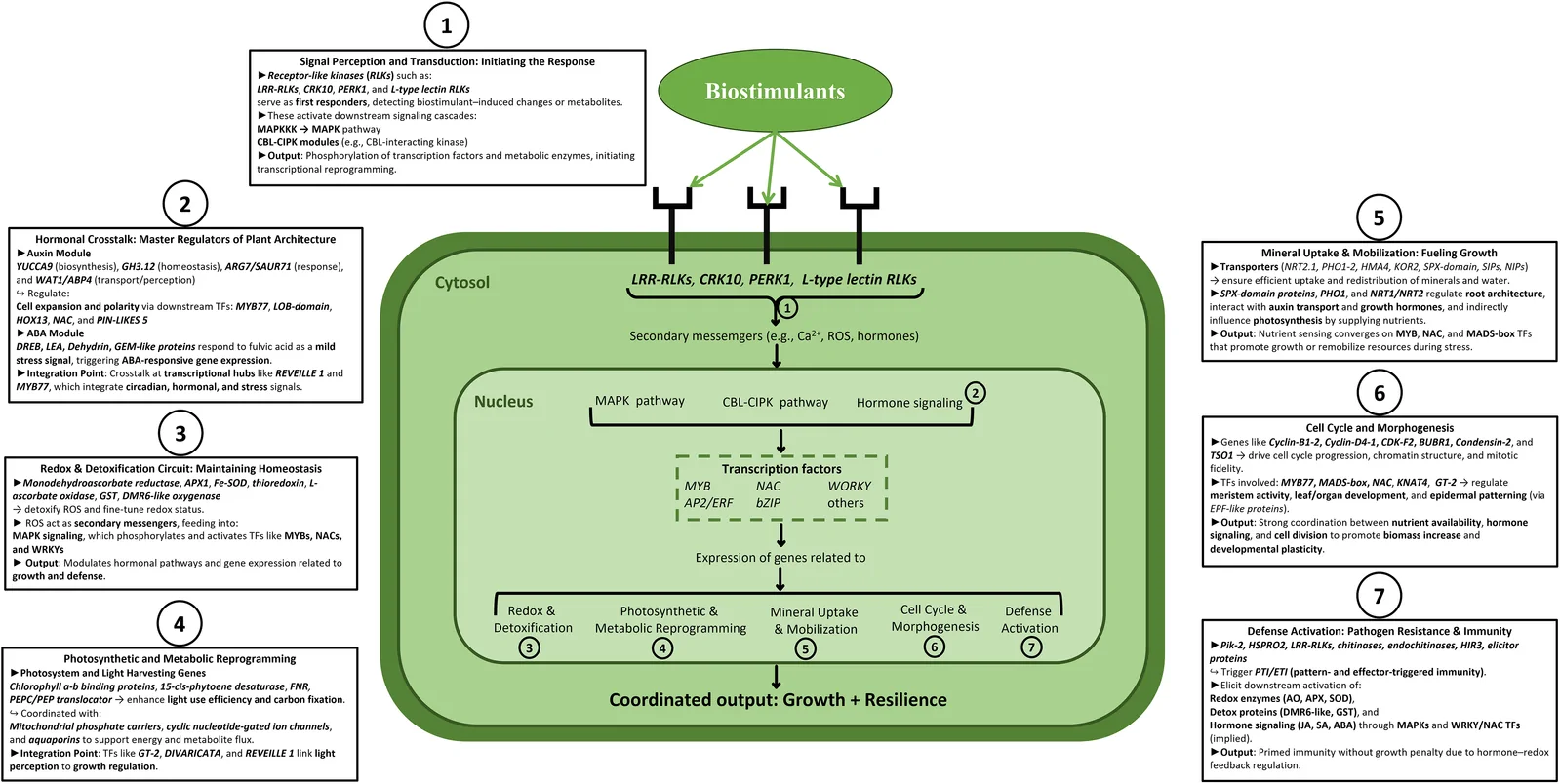
Conceptual model summarizing the multi-layered transcriptional and physiological responses of maize to the biostimulant treatments (BLG and BLGQ). The diagram illustrates seven interconnected modules that together describe how biostimulant perception and downstream signaling shape coordinated growth and resilience outcomes. (1) Signal perception and transduction: Receptor-like kinases (e.g., LRR-RLKs, CRK10, PERK1, L-type lectin RLKs) detect biostimulant-induced cues and activate MAPK and CBL–CIPK cascades. (2) Hormonal crosstalk: Auxin-, ABA-, and ethylene-associated genes integrate environmental cues into transcriptional reprogramming via TF families such as MYB, NAC, AP2/ERF, and LBD. (3) Redox and detoxification: Antioxidant enzymes and redox regulators maintain homeostasis and feed into ROS-dependent signaling pathways. (4) Photosynthetic and metabolic reprogramming: Chlorophyll-related genes, FNR, and PEP/PPR components enhance light capture, carbon fixation, and energy metabolism. (5) Mineral uptake and mobilization: Transporters (e.g., NRT, PHO1, SPX-domain proteins) support nutrient acquisition and redistribution, with downstream effects on growth-related TFs. (6) Cell cycle and morphogenesis: Genes involved in chromatin structure, cell division, and meristem regulation coordinate growth with nutrient availability and hormone signaling. (7) Defense activation: RLKs, PRRs, chitinases, and detoxification enzymes contribute to PTI/ETI-like immune activation without imposing strong penalties on growth. Together, these modules depict how BLG and BLGQ treatments converge on shared signaling hubs yet diverge in specific pathways, ultimately promoting balanced growth and stress resilience in maize.

Differential gene expression reflects changes in transcript abundance and does not necessarily indicate corresponding changes in protein abundance, enzyme activity, or physiological function. Furthermore, the differentially expressed genes identified in this study were not independently validated using quantitative RT-PCR or functional assays. Consequently, the biological roles of the identified candidate genes and regulatory pathways remain to be experimentally confirmed. Future studies integrating transcriptomic analyses with qRT-PCR validation, physiological measurements, metabolomics, proteomics, and functional characterization will be essential for establishing causal relationships between transcriptional responses and biostimulant-induced phenotypes.

## Conclusion

This study characterizes the temporal transcriptomic responses of maize to a combined formulation (BLGQ) of *Quillaja* saponin and fulvic acid, compared with both an untreated control and FA alone (BLG), thereby distinguishing the added effect of *Quillaja* saponin from the response elicited by FA alone. By comparing transcriptional response over time, at 3 h and 27 h, we demonstrate that the two formulations are associated with distinct patterns of differential gene expression, enriched biological pathways, and transcription factor activation, indicating stimulus- and time-dependent molecular responses. These results expand the current understanding of how maize transcriptionally responds to fulvic acid- and saponin-based biostimulants and provide a valuable resource of candidate genes and regulatory pathways for future investigation. As an exploratory transcriptomic study, our conclusions are limited to gene expression profiling, and functional validation of the identified candidate genes, together with integration of transcriptomics with proteomics, metabolomics, physiological measurements, and reverse genetics, will be necessary to establish the biological significance of these molecular signatures. Nevertheless, the transcriptomic framework presented here provides a foundation for future mechanistic studies and provides a transcriptomic resource that can support future mechanistic studies and the evidence-based evaluation and optimization of biostimulant formulations for sustainable crop production.

## Supporting information

S1 Table List of expressed genes in fulvic acid (BLG) vs. control treatments at early (3 hours) time point. (XLSX)

S2 Table List of differentially expressed genes in fulvic acid (BLG) vs. control treatments at early (3 hours) time point. (XLSX)

S3 Table List of expressed genes in mixture of fulvic acid and Quillaja extract (BLGQ) vs. control treatments at early (3 hours) time point. (XLSX)

S4 Table List of differentially expressed genes in mixture of fulvic acid and Quillaja extract (BLGQ) vs. control treatments at early (3 hours) time point. (XLSX)

S5 Table List of expressed genes in fulvic acid (BLG) vs. control treatments at late (27 hours) time point. (XLSX)

S6 Table List of differentially expressed genes in fulvic acid (BLG) vs. control treatments at late (27 hours) time point. (XLSX)

S7 Table List of expressed genes in mixture of fulvic acid and Quillaja extract (BLGQ) vs. control treatments at late (27 hours) time point. (XLSX)

S8 Table List of differentially expressed genes in mixture of fulvic acid and Quillaja extract (BLGQ) vs. control treatments at late (27 hours) time point. (XLSX)
